# Complete Genomic Sequence of the Potyvirus *Mashua Virus Y*, Obtained from a 33-Year-Old Mashua (*Tropaeaolum tuberosum*) Sample

**DOI:** 10.1128/MRA.01064-18

**Published:** 2018-10-18

**Authors:** Ian P. Adams, Adrian Fox, Neil Boonham, Roger A. C. Jones

**Affiliations:** a Fera, Sand Hutton, York, United Kingdom; b School of Natural and Environmental Sciences, Newcastle University, Newcastle upon Tyne, United Kingdom; c Institute of Agriculture, Faculty of Science, University of Western Australia, Crawley, Western Australia, Australia; d Department of Primary Industries and Regional Development, South Perth, Western Australia, Australia; Loyola University Chicago

## Abstract

We present the complete genomic sequence of a new potyvirus we tentatively call *Mashua virus Y* (MasVY), first isolated in 1984 from a plant of the Andean tuber crop mashua (*Tropaeolum tuberosum*, family *Tropaeolaceae*). There was a 70% nucleotide identity between MasVY and a genomic sequence of *Verbena virus Y*.

## ANNOUNCEMENT

In 1984, a tuber sample of mashua (*Tropaeolum tuberosum*, family *Tropaeolaceae*) (1) was obtained from an Andean root and tuber species collection at the Department of Applied Biology at the University of Cambridge in England. After the tuber was planted, a virus was transmitted from the plant grown to Nicotiana clevelandii plants. Both original mashua and inoculated N. clevelandii foliage developed mosaics. Electron microscopy of infected plant sap from N. clevelandii revealed approximately 750-nm-long flexuous filamentous potyvirus particles. In 1985, infected N. clevelandii leaf samples were freeze-dried in glass vials, which now are kept in the “Fera plant virus collection.” Mashua plants showing mosaic symptoms were first recorded in 1977 in Bolivia (2). In 1990, two potyviruses named Tropoaeolum 1 and 2 potyviruses were reported to cause leaf chlorosis in plants grown from mashua tubers imported to England from Peru (3). In 1998, a potyvirus infecting mashua imported to the United States from Ecuador was reported, and it was named Tropaeolum mosaic potyvirus (TropMV) (4). Whether TropMV resembles either of the Tropoaeolum 1 and 2 potyviruses is unclear. Subsequently, TropMV from Peruvian mashua was shown to reduce tuber yield (5). None of these mashua potyviruses were ever sequenced.

In 2017, using an RNAeasy kit (Qiagen, UK) including optional DNase treatment, total RNA was extracted from infected leaf material from N. clevelandii freeze-dried in 1985. An indexed plant ribosome-subtracted sequencing library was produced using the ScriptSeq complete plant leaf kit (Illumina, USA) following the manufacturer’s instructions. The indexed library was sequenced on a MiSeq instrument (Illumina) with a 600-cycle v3 kit. The resulting 748,695 paired reads were 3′ trimmed to a quality score of 20 with Sickle in paired-end mode (6) and assembled with Trinity v2 with the maximum memory allocation set to 99 GB of RAM, and the process allocated 64 central processing units (CPUs) (7), and the resulting contigs were compared to the GenBank nonredundant (nr) and nucleotide databases with BLAST+ (8). Reads of viral origin were extracted with the extract reads function in MEGAN (9). A 9,769-nucleotide (nt) contig was assembled by comparison with other genomes and constituted a complete potyvirus genome with a complete potyvirus coding region and typical 5′ and 3′ untranscribed regions (UTRs). The sequence contains a complete potyvirus coding region and typical 5′and 3′ UTRs. There was a 70% nt identity between the new genome and the nearest genomic sequence, *Verbena virus Y* (VVY) (GenBank accession number NC_010735), from the ornamental plant *Verbena × hybrid* (family *Verbenaceae*) reported from the United States (10). This <76% nt identity falls outside the species discrimination limit for *Potyviridae* (11). Establishing the relationship between the new potyvirus found in this study, which we tentatively name *Mashua virus Y* (MasVY), and the three mashua potyviruses named previously (3–5) awaits future studies. VVY belongs to an American subclade also containing *Pepper mottle virus* and *Potato virus Y* (10), so MasVY probably belongs with them. In 2018, to assess the likelihood of virus dissemination via Internet trading in unregulated plant species, RNA extracted from a mashua tuber obtained from a European country was sequenced as described above. The 1,335-nt potyvirus sequence recovered had 91% (nucleotide) and 96% (amino acid) coat protein identity with MasVY, which suggests MasVY’s presence in Internet-traded mashua tubers.

### Data availability.

The sequences described here were deposited in GenBank under accession numbers MH680824 (genome) and MH680823 (partial sequence). Raw data were deposited in the SRA under BioSample number SAMN10081141, which is part of BioProject PRJNA491634.
